# Adaptation of Self‐Esteem Stability Scale Into Turkish: Validity and Reliability Study in Nurses

**DOI:** 10.1002/nop2.70507

**Published:** 2026-04-03

**Authors:** Orkun Erkayiran, Fatma Demirkiran, Tarık Totan

**Affiliations:** ^1^ Department of Psychiatric Nursing, Faculty of Health Sciences Karamanoglu Mehmetbey University Karaman Türkiye; ^2^ Department of Mental Health Nursing, Faculty of Nursing Aydın Adnan Menderes University Aydın Türkiye; ^3^ Department of Educational Sciences, Department of Guidance and Psychological Counseling, Faculty of Education Aydın Adnan Menderes University Aydın Türkiye

**Keywords:** nurses, psychometrics, reliability, self‐concept, self‐esteem stability, validity

## Abstract

**Aim:**

This study aimed to adapt the Self‐Esteem Stability Scale (SESS) to Turkish and evaluate its validity and reliability among nurses. Understanding self‐esteem stability is crucial for assessing psychological well‐being in healthcare professionals.

**Design:**

A cross‐sectional methodological study.

**Methods:**

The study included 305 nurses (83% women, 17% men), aged 20–47 (mean age = 28.57), working in a hospital setting. The psychometric evaluation of the Turkish version of the SESS involved exploratory and confirmatory factor analyses (EFA and CFA), assessment of criterion validity, test–retest reliability, and internal consistency.

**Results:**

The Turkish SESS showed a clear one‐factor structure; EFA produced a first eigenvalue of 2.34 (> 1) explaining 78.08% of variance, with factor loadings 0.84–0.91 and corrected item–total correlations 0.66–0.78. CFA indicated excellent fit (CFI = 0.99; RMSEA = 0.03). Reliability was high (*α* = 0.86) with strong four‐week test–retest stability (*r* = 0.84).

**Conclusions:**

The Turkish version of the SESS is a valid and reliable tool for assessing self‐esteem stability among nurses.

**Patient or Public Contribution:**

No patient or public contribution.

## 1 Introduction

Nurses face unique challenges in their work environments, including high levels of stress, workplace violence, and difficult patient interactions. These stressors can negatively impact their mental and emotional well‐being, making self‐esteem an important factor in their professional performance. Self‐esteem, defined by Rosenberg (1979) as an individual's positive or negative attitude toward oneself, plays a critical role in nurses' resilience and job satisfaction (Kupcewicz and Jóźwik 2020). Research indicates that nurses with higher self‐esteem experience better mental health outcomes and are more resistant to burnout (Carson et al. 1997; Foster et al. 2018). As a recent example in nursing education, the Hindi adaptation of the Rosenberg Self‐Esteem Scale reported sound psychometrics among university nursing students (Aachal et al. 2024).

### 1.1 Importance of Self‐Esteem for Clinical Nurses

Self‐esteem plays a crucial role in various aspects of clinical nursing, impacting job satisfaction, job burnout, retention rates, and the quality of nursing care. Higher self‐esteem is positively associated with job satisfaction and fewer psychological problems, supporting better overall mental well‐being (Duran et al. 2021; Karanikola et al. 2007). It also buffers against burnout; both self‐esteem and self‐efficacy are negatively related to burnout, mitigating the effects of workload and stress (Molero et al. 2018; Nwafor et al. 2015).

In workforce outcomes, greater self‐esteem is linked to stronger retention intentions, and supportive practice environments further enhance both self‐esteem and retention (Cheng et al. 2020; Lee and Joo 2023; O'Malley et al. 2022). Importantly, self‐esteem influences service attitude and professional satisfaction, which directly contribute to the quality of nursing (Karanikola et al. 2007; Lou et al. 2011).

To strengthen nurses' self‐esteem, educational and organisational strategies are recommended. Curricula and in‐service training can incorporate self‐esteem–building components such as assertiveness, resilience, and interpersonal communication skills (Duran et al. 2021; Van Eckert et al. 2012). Organisations should provide mentoring and resilience‐building supports to help nurses cope with job demands (Bui et al. 2023; Henshall et al. 2023). Encouraging self‐care practices and supportive work climates can further enhance self‐esteem and overall well‐being, with benefits for retention and patient care (O'Malley et al. 2022; Williams et al. 2022).

Self‐esteem is a vital component for clinical nurses, influencing their job satisfaction, reducing burnout, increasing retention rates, and improving the quality of nursing care. Organisational and educational interventions aimed at enhancing self‐esteem can lead to significant benefits for both nurses and patients. While self‐esteem is generally stable, fluctuations may occur due to job‐related stressors like heavy workloads and lack of support (Mosadeghrad 2013). This variability in self‐esteem is conceptualised as self‐esteem stability, a concept gaining attention for its superior validity in predicting psychological adjustment compared to self‐esteem level alone (Kernis 2005; Okada 2010). Individuals with stable self‐esteem tend to have better mental health and overall life satisfaction (Butler et al. 1994; Kim and Cicchetti 2009).

Despite the importance of self‐esteem stability, no specific tool exists to measure it in Turkish. This study aims to adapt and validate the Self‐Esteem Stability Scale (Altmann and Roth 2018) for use among nurses in Turkey, addressing this gap in the literature.

## 2 Background and Conceptual Framework

The concept of self‐esteem stability has emerged as a vital construct in understanding individual differences in psychological resilience and adjustment. Unlike global self‐esteem, which represents an individual's overall self‐worth, self‐esteem stability refers to the temporal consistency of self‐esteem. Stable self‐esteem is associated with fewer fluctuations in self‐view and greater psychological well‐being (Kernis 2005). Conversely, unstable self‐esteem may lead to heightened emotional reactivity, vulnerability to stress, and poorer mental health outcomes (Okada 2010; Butler et al. 1994).

The Self‐Esteem Stability Scale (SESS) developed by Altmann and Roth (2018) measures this construct with superior specificity compared to traditional self‐esteem scales such as the Rosenberg Self‐Esteem Scale and Coopersmith Self‐Esteem Inventory. The adaptation and validation of the SESS for Turkish nurses offer an opportunity to investigate self‐esteem stability within a culturally relevant framework and contribute to the body of knowledge in nursing psychology.

Theoretical underpinnings for this study draw from Kernis's (2005) framework, which emphasises the role of self‐esteem stability in psychological resilience and adjustment. Additionally, cross‐cultural adaptation studies stress the need for methodological rigour in ensuring the validity and reliability of scales within diverse cultural settings (Hambleton et al. 2004; Polit and Beck 2017). This study's framework integrates these principles to provide a comprehensive approach to the adaptation and validation of the SESS.

The SESS is a unique scale that measures the stability of self‐esteem and is an important assessment tool, especially for nurses working under stress. While the Rosenberg Self‐Esteem Scale (RSES) measures general self‐esteem, the SESS offers a different perspective in understanding occupational resilience by assessing fluctuations in self‐esteem. While it is of great importance for nurses working in clinical settings that the scales are short and applicable, factor analyses show that self‐esteem stability can be assessed under a single factor. Unidimensional scales are more practical and time‐efficient than multidimensional scales and offer an effective assessment without creating unnecessary cognitive load for nurses. In the literature, it is emphasised that short and effective scales are more preferred for healthcare professionals. Short and effective scales are preferred in healthcare settings due to their time efficiency, ease of integration, cost‐effectiveness, and ability to maintain high reliability and validity. These attributes make them practical and valuable tools for healthcare professionals in both clinical and research contexts (Franke et al. 2013; Havenaar et al. 2004; Martinez‐Martin 2010; Monje and Sánchez‐Ferro 2024; Schroeders et al. 2016; Vittal and Barton 2016; Xiao et al. 2024).

## 3 Methods

### 3.1 Design

The present study is methodological research designed to test the validity and reliability of the Self‐Esteem Stability Scale (SESS).

### 3.2 Sample and Setting

Research data was collected in the Spring semester of 2019 from nurses working at the Hospital for Applications and Research of Aydın Adnan Menderes University. A total of 354 nurses were reached, and 325 nurses who volunteered participated, yielding a return rate of 91.80%. After excluding incomplete questionnaires, data from 305 nurses were included in the analysis. We justified N using widely cited factor‐analytic guidelines recommending subject‐to‐item ratios ≥ 5–10 and absolute Ns around ≥ 200 for stable EFA/CFA solutions (Comrey and Lee 1992; MacCallum et al. 1999; Mundfrom et al. 2005; Kline 2016; Hair et al. 2019). Our final sample (*N* = 305) exceeds these thresholds.

### 3.3 Data Collection

Face‐to‐face data collection was conducted in two stages. Initially, data were gathered from English language learners for pre‐validation. Subsequently, data were collected from nurses during their rest times to ensure convenience and reduce respondent burden.

### 3.4 Data Collection Tools

- Self‐Esteem Stability Scale (SESS): Developed by Altmann and Roth (2018), this scale evaluates the stability of self‐esteem over time. It comprises 3 items rated on a 6‐point Likert scale, with higher scores indicating greater stability. In this study, the internal consistency coefficient was 0.86.
- The Two‐Dimensional Self‐Esteem Scale (TDSES): The self‐report style is a 16‐item scale (Tafarodi and Swann 2001). A 16‐item self‐report scale was adapted into Turkish by Doğan (2011). The internal consistency coefficient for this study was 0.91.
- The Life Satisfaction Scale (LSS): A 5‐item scale rated on a 7‐point Likert scale (Diener et al. 1985). In this study, the internal consistency of the scale was determined to be 0.90 (Doğan and Totan 2013). The internal consistency coefficient for this study was 0.90.
- Judgmental Self‐Doubt Scale (JSDS): The Judgmental Self‐Doubt Scale was rated using a 6‐point Likert scale (Mirels et al. 2002). An 8‐item scale was adapted into Turkish by Akın et al. (2012). The internal consistency coefficient for this study was 0.94.
- The Subjective Happiness Scale (SHS): The Subjective Happiness Scale (SHS) is a 7‐point Likert‐type, self‐report measurement tool (Lyubomirsky and Lepper 1999). A Turkish adaptation of the scale was made (Doğan and Totan 2013). A 4‐item self‐report scale with an internal consistency coefficient of 0.73 for this study.
- The Willingness to Self‐Censor Scale (WSCS): The Willingness to Self‐Censor Scale (WSCS) is an 8‐item, one‐dimensional, 5‐point Likert‐type scale (Hayes et al. 2005a, 2005b). An 8‐item scale adapted into Turkish by Coskun et al. (2012). The internal consistency coefficient for this study was 0.77.
- The Psychological Well‐Being Scale (PWBS): The Psychological Well‐Being Scale aims to complement existing well‐being measurements and to measure socio‐psychological well‐being (Diener 2009). It has been adapted into Turkish (Telef 2013). A scale to measure socio‐psychological well‐being. The internal consistency coefficient for this study was 0.92.

### 3.5 Translation Process and Cultural, Linguistic Equivalence

The adaptation of the SESS involved forward translation, back‐translation, expert committee review, and pilot testing. The Turkish version was found to have a strong correlation with the original English version (*r* = 0.891, *p* < 0.001), ensuring linguistic and cultural equivalence.

### 3.6 Analysis

Data were analysed using IBM SPSS Statistics 25 and IBM SPSS Amos 23. Descriptive statistics determined means and standard deviations. Reliability and validity were assessed using Exploratory Factor Analysis (EFA), Confirmatory Factor Analysis (CFA), Cronbach's alpha, and item‐total correlations. The decision to conduct EFA was based on the need to understand the factor structure within the Turkish cultural context. CFA was subsequently performed to confirm the findings. Because recruiting an independent validation sample of nurses was not feasible during the study period, both EFA and CFA were conducted on the same dataset (*n* = 305). To mitigate the risk of capitalizing on chance, we determined the factor number using multiple criteria (eigenvalues and scree plot) and evaluated model adequacy with commonly reported fit indices (CFI, RMSEA), alongside internal consistency and test–retest stability.

### 3.7 Ethical Considerations

Prior to embarking upon the research, the scale owner was contacted via email to request permission to utilise the scale. The ethical approval for the study was obtained from the Non‐Invasive Clinical Research Ethics Committee of the Faculty of Nursing at Aydin Adnan Menderes University on February 18, 2019 with protocol number 2019/068. The institution's permission for the study was obtained from the Office of the Rector at Aydin Adnan Menderes University and the Head Office of the Hospital for Applications and Research on April 09, 2019 with file number 23118, and oral consent was obtained from the nurses during data collection. In order to proceed with the translation phase of the study, the relevant deanery was approached and permission was duly granted.

## 4 Results

### 4.1 Sociodemographic Characteristics of Participants

Out of the total participants in the study, 83% were female nurses (*n* = 253) and 17% were male nurses (*n* = 52). The age range of the participants was between 20 and 47 years, with an average age of 28.57 ± 5.57. Table 1 shows the distribution of the participants in terms of their age, education, gender, marital status, and units they work in (Table 1).

**TABLE 1:** Sociodemographic characteristics of nurses.

	*n*	%
Gender		
Female	253	83
Male	52	17
Marital status		
Married	151	49.5
Single	154	50.5
Education status		
High school	41	13.4
Associate degree	22	7.2
Bachelor's degree	210	68.9
Graduate degree	32	10.5
Years of experience in profession	Mean	SD
	7.00	5.154
1–5 years	145	47.5
6–10 years	99	32.5
11–15 years	40	13.1
16–20 years	11	3.6
21–25 years	10	3.3
Choosing a profession voluntarily		
Yes	214	70.2
No	91	29.8
Satisfaction with profession		
Not at all satisfied	8	2.6
A little dissatisfied	15	4.9
Satisfied	130	42.6
A little satisfied	112	36.7
Very satisfied	40	13.1
Total	305	100.0

### 4.2 Construct Validity and Item Analysis

EFA and CFA were performed on the same dataset (*n* = 305) due to feasibility constraints.

#### 4.2.1 Exploratory Factor Analysis (EFA)

The suitability of the data for factor analysis was examined using the Kaiser‐Meyer‐Olkin (KMO) coefficient and Bartlett's Test of Sphericity. It is established that for factor analysis, the KMO coefficient must be above 0.50, and Bartlett's test must be significant at the 0.05 level (Çokluk et al. 2016; Field 2013; Leech et al. 2013). The KMO coefficient of 0.70 and a significant Bartlett's Test result (*χ* ^2^ (3) = 444.520, *p* < 0.001) demonstrated the data's suitability for factor analysis.

**FIGURE 1:**
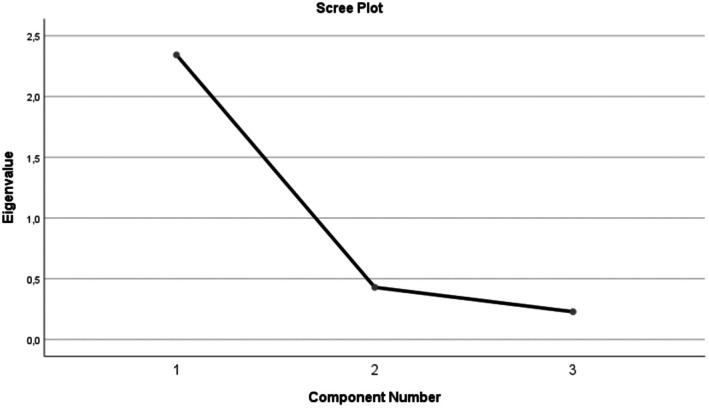
Scree plot of eigenvalues (PCA).

The Exploratory Factor Analysis (EFA) revealed a one‐factor structure explaining 78.08% of the total variance. Factor loadings ranged 0.84–0.91 and corrected item–total correlations 0.66–0.78 (Table 2). The first eigenvalue was 2.342 (> 1), and subsequent eigenvalues were < 1 (0.429, 0.228); the scree plot showed a clear elbow after Component 1, consistent with a single factor (Table 3; Figure 1). This finding aligns with the criterion that in one‐factor scales, an explained variance of 30% or more is considered sufficient (Büyüköztürk 2007). These results indicate high internal consistency and that the items effectively measure the same construct.

**TABLE 2:** SESS factor loads and item‐total correlations.

Items		Factor loads	Item‐total correlation
1.	My attitude toward myself never changes.	0.84	0.66
2.	When I compare myself with others, my evaluations of my abilities often change.^a^	0.90	0.76
3.	My positive and negative feelings about myself often get mixed together.^a^	0.91	0.78

^a^ The items are reverse‐coded.

**TABLE 3:** Eigenvalues and variance explained (PCA).

Component	Eigenvalue	% of Variance	Cumulative %
1	**2.342**	**78.08**	**78.08**
2	0.429	14.31	92.39
3	0.228	7.62	100.00

*Note:* Extraction method: Principal Component Analysis; rotation: none. Bold values indicate the retained component with an eigenvalue > 1.

#### 4.2.2 Confirmatory Factor Analysis (CFA)

Confirmatory Factor Analysis (CFA) was performed to validate the one‐factor structure identified in the EFA using the maximum likelihood estimation method. Model fit was assessed using various indices: a chi‐square/df ratio (*χ* ^2^/df) below 5 indicates an acceptable fit, while values below 3 suggest a good fit (Kline 2011). Other fit indices include the Comparative Fit Index (CFI), Incremental Fit Index (IFI), Relative Fit Index (RFI), and Normed Fit Index (NFI), where values above 0.95 indicate a good fit. For the Root Mean Square Error of Approximation (RMSEA) and Standardized Root Mean Square Residual (SRMR), values below 0.05 indicate a good fit (Hoyle 1995; Hu and Bentler 1999).

The CFA results confirmed the one‐factor structure, with fit indices as follows: *χ* ^2^/df (1.73/1) = 1.73, NFI = 0.99, CFI = 0.99, IFI = 0.99, RFI = 0.98, GFI = 0.99, AGFI = 0.98, RMSEA = 0.026, and SRMR = 0.06. These results demonstrate that the scale's structure is valid in the Turkish sample. Standardized factor loadings and R^2^ values are presented in Table 4.

**TABLE 4:** Factor loadings and parameter estimates related to confirmatory factor analysis.

Items	Standardized loadings	*R* ^2^	SE	*z*	*p*
Item 1	0.67	0.45	0.059	11.355	0.000*
Item 2	0.85	0.73	0.043	19.767	0.000*
Item 3	0.89	0.80	0.036	24.722	0.000*

* *p* < 0.001.

### 4.3 Criterion‐Related Validity

For criterion‐related validity, the Self‐Esteem Stability Scale (SESS) was correlated with the Two‐Dimensional Self‐Esteem Scale (TDSES), Judgmental Self‐Doubt Scale (JSDS), Life Satisfaction Scale (LSS), Subjective Happiness Scale (SHS), Psychological Well‐Being Scale (PWBS), and the Willingness to Self‐Censor Scale (WSCS). Significant positive and negative relationships between SESS and these scales support its validity (Table 5).

**TABLE 5:** Correlation results for criterion‐related validity.

		TDSES	JSDS	LSS	SHS	PWBS	WSCS
SESS	*r*	0.57	−0.43	0.60	0.67	0.70	−0.54
	*p*	0.000*	0.000*	0.000*	0.000*	0.000*	0.000*

* *p* < 0.001.

### 4.4 Reliability

Cronbach's alpha coefficient was calculated for internal consistency, yielding a value of 0.86. This meets the reliability threshold of 0.70 (Büyüköztürk 2007), confirming the scale's internal consistency.

### 4.5 Test–Retest Reliability

Data from 305 nurses were initially collected, and 96 nurses completed the scale again after 4 weeks. The Pearson correlation coefficient between the two measurements was 0.84 (*p* < 0.001), indicating high test–retest reliability.

## 5 Discussion

The findings of this study demonstrate the psychometric properties of the Self‐Esteem Stability Scale (SESS), a one‐dimensional scale developed by Altmann and Roth (2018), and its successful adaptation to Turkish. The scale is valid and reliable for measuring self‐esteem stability in Turkish populations, particularly among nurses.

The one‐factor structure of the scale, explaining 78.08% of the total variance, is consistent with the original scale's findings and meets the criteria for robustness. Reliability analyses, including Cronbach's alpha and test–retest results, confirmed the scale's reliability. Criterion‐related validity analyses revealed significant relationships between the SESS and related constructs, supporting its use in assessing self‐esteem stability.

Self‐esteem stability reflects the extent to which an individual's self‐esteem fluctuates over time. Unlike self‐esteem level, which measures self‐worth at a given point, stability captures the consistency of these evaluations. This distinction is critical in understanding how fluctuations in self‐esteem influence psychological well‐being. For instance, previous studies (e.g., Kernis 2005) have linked unstable self‐esteem to increased vulnerability to emotional distress. The results of this study align with such findings and highlight the importance of considering stability as a distinct construct in research and practice.

### 5.1 Comparison With Similar Scales

The SESS complements other self‐esteem measures used in Turkey, such as the Rosenberg Self‐Esteem Scale (RSES). While the RSES measures self‐esteem level, the SESS focuses on its stability, offering a nuanced understanding of self‐esteem dynamics. Studies validating the RSES in Turkish populations (Doğan 2011; Korkmaz 2022) support the robustness of self‐esteem measurements across cultural contexts. The SESS's strong psychometric properties further contribute to this body of research.

### 5.2 Limitations and Future Research

This study has two key limitations. The sample was confined to university hospital nurses, and both the EFA and CFA were conducted on the same dataset, which may inflate model fit and increase the risk of sample‐specific findings. We sought to mitigate this by applying multiple factor‐retention criteria, reporting comprehensive fit indices, and providing additional reliability/validity evidence (internal consistency, test–retest stability, convergent validity).

Future studies should replicate the factor structure in independent samples across hospitals and settings and evaluate the SESS's applicability in diverse populations and professions. Longitudinal designs are needed to examine causal relationships between self‐esteem stability and outcomes such as burnout, job satisfaction, and patient‐care quality. Incorporating scenarios or symbols into the SESS could also improve respondents' ability to reflect on self‐esteem fluctuations.

### 5.3 Relevance to Nursing Practice, Education, and Research

The findings of this study have significant implications for nursing practice, education, and research. In practice, the SESS can be used to identify nurses at risk of emotional distress due to unstable self‐esteem. Targeted interventions, such as resilience training programs, could enhance nurses' psychological well‐being and professional performance.

In education, integrating self‐esteem stability concepts into nursing curricula can foster self‐awareness and emotional regulation skills among students. Workshops on self‐esteem management, particularly during clinical training, could better prepare future nurses for the challenges of their profession.

In research, the SESS provides a validated tool for studying self‐esteem stability in Turkish healthcare professionals. Future studies could explore its impact on key outcomes such as job satisfaction, burnout, and mental health. Expanding research to other professions and cultural contexts could provide valuable comparative insights.

By addressing self‐esteem stability, this study contributes to a deeper understanding of nurses' mental health and offers practical strategies for improving their resilience and well‐being.

## 6 Conclusion

The adaptation and validation of the Self‐Esteem Stability Scale (SESS) for Turkish populations, specifically nurses, represents a meaningful contribution to the field of psychological assessment. This study highlights the scale's robust psychometric properties, including its one‐factor structure, strong internal consistency, and significant criterion‐related validity.

Understanding self‐esteem stability provides critical insights into how fluctuations in self‐worth affect individuals' emotional well‐being and professional functioning. The findings emphasise the importance of incorporating stability measures into both research and practical applications within nursing. As a validated tool, the SESS can aid in identifying individuals at risk for emotional distress and guide interventions aimed at enhancing self‐esteem consistency.

Future research should continue exploring the scale's application across diverse cultural and professional contexts, while also investigating longitudinal relationships between self‐esteem stability and key psychological or occupational outcomes. By fostering a deeper understanding of self‐esteem dynamics, this research paves the way for targeted strategies that support the mental health and professional resilience of healthcare professionals.

## Author Contributions

Research Planning: O.E., F.D., T.T.; Data Collection: O.E.; Data Analysis: O.E., T.T.; The Preparation of the Research Report: O.E., F.D. and T.T. This study was presented as an oral presentation at VI. International, X. National Psychiatric Nursing Congress, 20–23 October 2021.

## Funding

The authors have nothing to report.

## Ethics Statement

Prior to embarking upon the research, the scale owner was contacted via email, and permission to utilise the scale was obtained. In order to proceed with the translation phase of the study, the relevant deanery was approached, and permission was duly granted. Ethical approval for this study was obtained from the Non‐Invasive Clinical Research Ethics Committee of the Faculty of Nursing at Aydın Adnan Menderes University on February 18, 2019 (Protocol number: 2019/068). Institutional permission was obtained from the Office of the Rector at Aydın Adnan Menderes University and the Head Office of the Hospital for Applications and Research on April 09, 2019 (File number: 23118). Oral informed consent was obtained from all participating nurses during data collection.

## Conflicts of Interest

The authors declare no conflicts of interest.

## Data Availability

The data that support the findings of this study are available from the corresponding author upon reasonable request.
